# Sexual dysfunction in women with breast cancer of Northeast Brazil: a retrospective longitudinal study

**DOI:** 10.1038/s41598-023-47684-7

**Published:** 2023-11-22

**Authors:** Thais Sousa Rodrigues Guedes, Marcello Barbosa Otoni Gonçalves Guedes, Johnnatas Mikael Lopes, Rebeca de Castro Santana, Jamily Borba de Vasconcelos, Eva Regina de Medeiros, Vitor Leandro da Cunha, Amanda Almeida Gomes Dantas, Javier Jerez-Roig, Dyego Leandro Bezerra de Souza

**Affiliations:** 1 Graduate Program in Health Science, Federal University of Rio Grande do Norte (UFRN), Natal, Rio Grande do Norte Brazil; 2 Department of Physical Therapy, Federal University of Rio Grande do Norte (UFRN), Natal, Rio Grande do Norte Brazil; 3 Federal University of Vale do São Francisco (UNIVASF), Paulo Afonso, Bahia Brazil; 4 Research Group on Methodology, Methods, Models, and Outcomes of Health and Social Sciences (M3O), Faculty of Health Sciences and Welfare, Center for Health and Social Care Research (CESS), University of Vic-Central University of Catalonia (UVic-UCC), C. Sagrada Família, 7, 08500 Vic, Spain; 5 Institute for Research and Innovation in Life Sciences and Health in Central Catalonia (IRIS-CC), Vic, Spain; 6 Department of Public Health, Graduate Program in Health Science, Federal University of Rio Grande do Norte (UFRN), Natal, Rio Grande do Norte Brazil

**Keywords:** Cancer, Breast cancer

## Abstract

Breast cancer treatment leads to physical and psychological changes. The aim of this study was to analyze the incidence of sexual dysfunction and its risk factors in women diagnosed with breast cancer. This retrospective cohort study included women diagnosed and treated for breast cancer (exposed group, n = 90) and healthy women (non-exposed group, n = 93). Data were collected from February 2019 to October 2021 in the state of Rio Grande do Norte (Northeast Brazil), from medical records and using the Female Sexual Function Index (FSFI) questionnaire. Data were collected from medical records and using the Female Sexual Function Index (FSFI) questionnaire. Primary outcomes were analyzed using binary logistic regression. The Mann–Whitney test was used to analyze FSFI domains between groups. The exposed group had a 74% incidence of sexual dysfunction and 3.9 times increased chances of having sexual dysfunction compared with the non-exposed group (OR 3.9, CI 1.8 to 8.2, *p* < 0.001). Presence of comorbidities increased the chances of sexual dysfunction by 2.5 times (OR 2.5, CI 1.2 to 4.9, *p* = 0.009). Women diagnosed and treated for breast cancer had a higher incidence of sexual dysfunction than healthy women. Furthermore, comorbidities also increased the chances of sexual dysfunction regardless of exposure to breast cancer.

## Introduction

Breast cancer ranks first as the most common cancer among women and has high incidence and mortality in low- to high-income countries. In Brazil, 110,000 new cases are estimated for 2030^1^. The prognosis for breast cancer is good when diagnosed early. For example, the 5-year relative survival rate in the United States Canada, and Australia is 90%, whereas in Brazil it is 72.9%^1,2^.

Common breast cancer treatments, including surgery, chemotherapy, radiotherapy, and hormone therapy, may result in induced menopause and decreased vaginal lubrication, affecting sexual excitement and desire^3,4^. Moreover, changes in body image^5^ may also impact sexual function, modifying femininity and sexual identity^6^. Sexual dysfunction is a disorder of sexual desire and encompasses psychophysiological changes in sexual desire, sexual excitement, orgasm, and satisfaction, leading to interpersonal difficulties and suffering^7^.

Cross-sectional studies conducted with healthy women indicate a 25% to 50% prevalence of sexual dysfunction.^7–12^ However, up to 75% of women diagnosed with breast cancer report persistent challenges in their sexual life due to the long-term effects of cancer treatment^13–16^.

Assessing sexual dysfunction contributes to understanding the experience of cancer diagnosis and its treatment, elucidating impacts on self-esteem and social participation^17,18^. Therefore, this study aimed to analyze the incidence of sexual dysfunction and its risk factors in women diagnosed and treated for breast cancer living in Northeast Brazil.

## Methods

### Study design

This study followed a retrospective cohort design^19^, conducted in accordance with the Strengthening the Reporting of Observational studies in Epidemiology. Groups were formed primarily by identifying the cohort highly exposed to the phenomenon of interest (i.e., breast cancer) and secondarily by selecting the comparison group (i.e., healthy women).

The exposed group consisted of women diagnosed with breast cancer (C-50) according to the International Classification of Diseases (ICD-10) and submitted to one or more oncological treatments (surgery, chemotherapy, radiotherapy, or hormone therapy) between 2013 and 2017 in Natal, Rio Grande do Norte, Brazil that were retrospectively identified and researched 12 months or up to 8 years after cancer diagnosis. The non-exposed group consisted of healthy women from the community.

For sample size calculation, a prevalence of 75% of sexual dysfunction was considered for the exposed group^13,15^ and 50% for the non-exposed group^7,9^. The relative risk was considered as 1.5, with a significance level of 5% and statistical power of 80%, resulting in 116 women in the study, 58 in each group^19^.

### Inclusion and exclusion criteria

For the exposed group, inclusion criteria were women diagnosed with breast cancer for at least 12 months, submitted to cancer treatment, and undergoing clinical follow-up, and exclusion criteria were women with intellectual disabilities or unable to understand the questionnaires, who presented cancer recurrences, were under treatment or palliative care, who had sexual dysfunction before the breast cancer diagnosis, or who had debilitating or disabling morbidities not associated with breast cancer and its treatment. The non-exposed group consisted of healthy women without any type of cancer, ideally companions of the participants from the exposed group (i.e., friends, family members, and caregivers), and exclusion criteria were women with intellectual disabilities or unable to understand the questionnaires. Participants were matched by marital status and education level.

### Data collection

Data were collected from February 2019 to October 2021 at a non-profit care units for patients with breast cancer (Liga Norte Riograndense Against Cancer, Natal, Rio Grande do Norte, Brazil) and via telephone due to the Covid-19 pandemic.

Data of the exposed group were collected using medical records and transferred to a digital form including sociodemographic information (name, age, body mass index, race, marital status, educational level, income according to Brazilian minimum wage in 2017, and access to health services); life habits (tobacco and alcohol consumption and physical activity frequency); gynecological and obstetric history (pregnancies, deliveries, abortions, breastfeeding, nulliparity, gynecological appointments and pap smear exam frequency, menopausal status, hormonal replacement, mammography, breast self-examination, and family and personal history of cancer); sexual activity (investigated with the questions “are you sexually active?” and “did you have sexual activity before treatment for breast cancer?”); comorbidities (hypertension, diabetes, arthritis, arthrosis, dyslipidemia, and depression); use of medications; and clinical variables (diagnosis, staging, type and duration of treatment, and postoperative complications).

Four trained researchers contacted the participants in-person (before Covid-19 pandemic) or via telephone (during Covid-19 pandemic). Similar data were collected from the non-exposed group, excluding information on cancer and its treatments.

Women from both groups were classified according to the presence or absence of sexual dysfunction based on the Female Sexual Function Index (FSFI) questionnaire. The FSFI was translated and validated for Brazilian Portuguese by Thiel et al. (2008)^20^. The questionnaire evaluates sexual functioning in the domains of desire, excitement or arousal, vaginal lubrication, orgasm, satisfaction, and pain. The score is based on a 6-point Likert scale where the total score is the sum of each domain multiplied by a correction value. A cutoff point of ≤ 26 was proposed by Wiegel et al. (2005) to classify the presence of sexual dysfunction. The FSFI presented high test–retest reliability and internal consistency^21^.

The study was approved by the committee for ethics in research with humans of the Liga Norte Riograndense Against Cancer (no 2.197.925). All participants signed the informed consent form, and for illiterate participants, we obtained informed consent from parents/or legal guardians.

### Data analysis

The primary outcome encompassed sexual dysfunction and was analyzed as a dichotomous variable, showing a binomial distribution. We opted for a binary logistic regression also due to the retrospective characteristic of data. The significance level of independent variables was *p* ≤ 0.20 in the gross analysis and added to the final equation according to the theoretical model. The best-adjusted model was chosen using its significance and the lowest value of the Akaike Information Criterion (AIC). The Chi-square test assessed the independent variables for the primary outcome and the effect of measure was the odds ratio (OR). The non-parametric Mann–Whitney test and descriptive analysis were performed for analyzing the FSFI domains between groups. A significance level of 5% was considered in all analyses to minimize possible type I errors. Software SPSS version 20.0 was utilized for statistical analysis of data.

### Ethical approval and consent to participate

The study was approved by the committee for ethics in research with humans of the Liga Norte Riograndense Against Cancer (no 2.197.925).

## Results

### Sample

**Figure 1:**
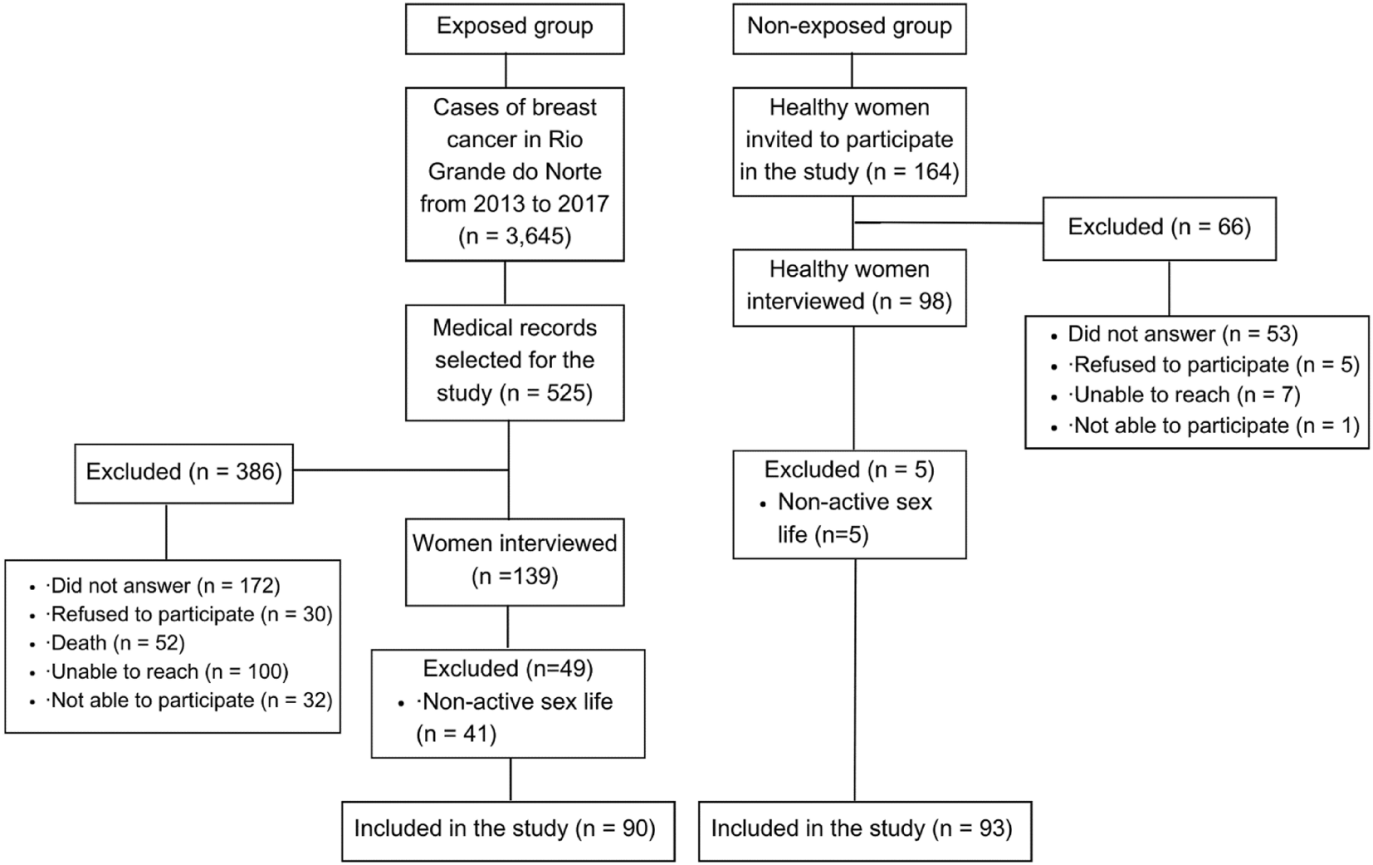
Flowchart of the exposed and non-exposed groups.

A total of 3645 new cases of breast cancer were registered from 2013 to 2017 (869 in 2013, 857 in 2014, 624 in 2015, 678 in 2016, and 617 in 2017). The first phase of this study encompassed collecting data from medical records of women under clinical follow-up to recruit for inclusion in the exposed group. Women were contacted by telephone and invited to participate in the study. Of 525 women, 386 were not included in the study. The remaining 139 women were interviewed, and 90 were included. For the non-exposed group, 164 women were invited to participate in the study, 98 were interviewed, and 93 were included. Reasons for not participating in the study are shown in Fig. 1.

### Sociodemographic data, life habits, and gynecological, obstetric, and clinical history

Ninety women were included in the exposed group, whereas 93 healthy women composed the non-exposed group (Table 1). The sample was predominantly white or brown, married, and catholic, with complete high school or higher education, and had an income up to one or two to five minimum wages. Most participants performed physical activity and did not smoke or use alcohol. Also, the presence of comorbidities was high in both groups, and BMI indicated overweight in the non-exposed group and obesity in the exposed group. Most participants in the exposed group reported being in menopausal. Reports of good health and satisfaction with body image were frequent. Lastly, most participants denied depression diagnosis. Clinical data of the exposed group indicated that conservative surgery was the most prevalent as a loco-regional treatment, and adjuvant treatment was indicated.

**Table 1:** Characterization of the sample.

	Exposed group		Non-exposed group		*p*-value
	N (90)	%	N (93)	%	
Mean age					
≥ 51 years	56	62.2	23	24.7	0.001*^a^
≤ 50 years	34	37.8	70	75.3	
Race					
Yellow	1	1.1	4	4.3	0.014*^a^
Black	13	14.4	4	4.3	
Brown	28	31.1	44	47.3	
White	48	53.3	41	44.1	
Religion					
Other	7	7.8	10	10.8	0.223^a^
Spiritist	1	1.1	3	3.2	
Evangelical/Protestant	17	18.9	26	28.0	
Catholic	65	72.2	54	58.1	
Marital status					
Married or common-law marriage	71	78.9	80	86.0	0.245^a^
Other (single/widowed/divorced)	19	21.1	13	14.0	
Educational level					
Higher education	38	42.2	47	50.5	0.227^a^
High school	32	35.6	34	36.6	
Illiterate or Elementary school	20	22.2	12	12.9	
Smoking status					
Yes	22	24.4	8	8.6	0.005*^a^
No	68	75.6	85	91.4	
Alcohol use					
Yes	27	30.0	41	44.1	0.066^a^
No	63	70.0	52	55.9	
Income (in number of minimum wages**)					
0–1	32	35.6	21	22.6	0.001*^b^
1–2	22	24.4	15	16.1	
2–5	21	23.3	28	30.1	
5–10	10	11.1	23	24.7	
10–20	5	5.6	4	4.3	
≥ 20	–		2	2.2	
Comorbidities					
Yes	47	52.2	37	39.8	0.104^a^
No	43	47.8	56	60.2	
Physical activity					
Yes	58	64.4	57	61.3	0.760^a^
No	32	35.6	36	38.7	
Nulliparity					
Yes	57	63.3	55	59.1	0.442^a^
No	30	33.3	38	40.9	
N/A	3	3.4	–		
Breastfeeding					
No	10	11.1	17	18.3	0.212^a^
Yes	80	88.9	76	81.7	
Menopause					
No	19	21.1	66	71.0	0,001*^a^
Yes	71	78.9	27	29.0	
Hormone replacement					
Yes	13	14.4	2	2,2	0.047*^a^
No	71	78.9	50	53.8	
N/A	6	6.7	41	44.1	
Depression					
Yes	16	17.8	16	17.2	0.846^a^
No	69	76.7	77	82.8	
N/A	5	5.6	–		
Body image satisfaction					
Very satisfied	14	15.6	2	2.2	0.023*^a^
Satisfied	36	40.0	23	24.7	
Moderately satisfied	27	30.0	19	20.4	
A little satisfied	11	12.2	12	12.9	
Unsatisfied	2	2.2	1	1.1	
N/A	–	–	36	38.7	
Self-perceived health					
Very poor	2	2.2	1	1.1	0.001*^a^
Poor	11	12.2	3	3.2	
Good	36	40.0	56	60.2	
Very good	2	2.2	19	20.4	
Excellent	3	3.3	5	5.4	
N/A	36	40.0	9	9.7	
Sexual dysfunction					
No	21	23.3	56	60.2	0.001*^a^
Yes	67	74.4	37	39.8	
N/A	2	2.2	–	–	
Surgery type					
Mastectomy	43	47.8			
Conservative	46	51.1			
N/A	1	1.1			
Chemotherapy					
Yes	67	74.4			
No	23	25.6			
Radiotherapy					
Yes	79	87.8			
No	11	12.2			
Hormone therapy					
Yes	28	31.1			
No	57	63.3			
N/A	5	5.6			

^a^Chi-squared test; ^b^Fisher exact test. *p ≤ 0.05. N/A: does not apply or did not answer.

**Minimum wage in 2017 was R$937 (approximately USD300).

The mean age for the first sexual intercourse was 20.7 (± 3.8) in the exposed group and 20.1 (± 3.9) in the non-exposed group. The number of pregnancies and deliveries was 2.6 (± 1.4) and 2.2 (± 1.1) in the exposed group and 1.9 (± 1.1) and 2.0 (± 3.2) in the non-exposed group, respectively.

### Sexual function

Incidence of sexual dysfunction was 74% in the exposed group, and 39% in the non-exposed group. Gross analysis indicated that the exposed group had 4.8 (2.5 to 9.1) times the chances of sexual dysfunction than the non-exposed group. The chances of sexual dysfunction increased by 0.6% for each added year of age and 36% for each pregnancy. Moreover, the chances of sexual dysfunction increased in 2.9 (1.5 to 5.4) times with having comorbidities, 4.8 (1.2 to 18.5) times when participants self-perceived their health as poor or very poor, and 3.0 (1.2 to 7.4) times when smoking. Otherwise, menopause and income higher than one minimum wage reduced the sexual dysfunction by 0.71 and 0.55 times, respectively (Table 2).

**Table 2:** Gross and final adjusted model for sexual dysfunction in the exposed and non-exposed groups.

	Gross model			Final adjusted model		
	OR	95% CI	P value	OR	95% CI	P value
Group						
Exposed	4.82	2.54 to 9.18	0.001*	3.92	1.88–8.20	0.001*
Non-exposed	1			1		
Menopause						
Yes	0.29	0.15 to 0.53	0.001*	1.43	0.60–3.41	0.413
No	1			1		
Comorbidity						
Yes	2.92	1.58 to 5.42	0.001*	2.51	1.26–4.99	0.009*
No	1			1		
Age						
≥ 51 years	2.67	1.44 to 4.96	0.002*	1.02	0.43–2.39	0.962
≤ 50 years	1			1		
Self-perceived health						
Poor or very poor	4.87	1.27 to 18.57	0.020*			
Good	1.56	0.67 to 3.61	0.293			
Excellent or very good	1					
Smoking status						
Yes	3.04	1.24 to 7.47	0.015*			
No	1					
Income (minimum wage**)						
≥ 1	0.45	0.22 to 0.91	0.027*			
˂ 1	1					
Pregnancies	1.36	1.06 to 1.76	0.016*			
Nulliparity						
Yes	0.66	0.35 to 1.23	0.194			
No	1					
Breastfeeding						
No	0.67	0.30 to 1.51	0.344			
Yes	1					
Body image satisfaction						
Satisfied or very satisfied	0.96	0.26 to 3.45	0.954			
Little or moderately satisfied	0.70	0.27 to 1.76	0.448			
Unsatisfied	1					
Race						
Other	0.68	0.37 to 1.23	0.205			
White	1					
Religion						
Non-catholic	0.60	0.32 to 1.10	0.103			
Catholic	1					
Alcoholic						
Yes	0.58	0.31 to 1.06	0.080			
No	1					
Hormone replacement						
Yes	0.35	0.09 to 1.29	0.116			
No	1					
Age at first sexual intercourse	1.03	0.95 to 1.11	0.442			
BMI	1.03	0.97 to 1.10	0.281			
Deliveries	0.98	0.87 to 1.10	0.792			
Abortions	0.90	0.58 to 1.42	0.676			

BMI: body mass index, CI: confidence interval, OR: odds ratio, *p ≤ 0.05.

** Minimum wage in 2017 was R$937 (approximately USD300).

Adjusted analysis indicated that cancer and comorbidities significantly increased by 3.9 (1.8 to 8.2) and 2.5 (1.2 to 4.9) times the chances of sexual dysfunction among the exposed group compared to the non-exposed group. We used a model that suggests a correlation between exposure to cancer and the development of sexual dysfunction. The model was adjusted considering menopause, self-perceived health, smoking status, pregnancy, and age variables. However, the final model considered only menopause and age (Table 2).

The non-exposed group scored higher in all domains of the FSFI, indicating better sexual functioning (Table 3).

**Table 3:** Analysis of the Female Sexual Function Index domains for the exposed and non-exposed groups.

Domains	Exposed group (n = 90)	Non-exposed group (n = 93)	*P* value
	Mean (SD)	Mean (SD)	
Sexual desire	3.1 (± 1.7)	3.8 (± 0.9)	0.001*
Sexual excitement/arousal	3.6 (± 1.6)	4.5 (± 0.9)	0.001*
Vaginal lubrication	3.2 (± 0.7)	3.4 (± 0.4)	0.002*
Orgasm	3.5 (± 0.8)	3.8 (± 0.6)	0.008*
Sexual satisfaction	4.6 (± 1.4)	5.2 (± 0.8)	0.005*
Pain	4.6 (± 1.5)	5.3 (± 0.8)	0.007*
Total	22.8 (± 5.1)	26.3 (± 3.2)	0.001*

SD: standard deviation; *p ≤ 0.05.

## Discussion

This study investigated sexual dysfunction in women diagnosed and treated for breast cancer. Our results showed a high incidence of sexual dysfunction among this population. We observed that exposure to breast cancer and comorbidities increased the chances of sexual dysfunctions, regardless of menopause and age. Moreover, the exposed group had a worse sexual function in all domains assessed by the Female Sexual Function Index. Our findings corroborate previous studies and highlight the importance of periodically assessing the sexual function of women diagnosed with breast cancer and under treatment^22–24^.

A recent systematic review showed an incidence of 30% to 80% of sexual dysfunction in women with cervical, gynecological, and breast cancer ^22^. Furthermore, regardless of the category of dysfunction, women had a 2.7 to 3.5 times greater risk of developing sexual dysfunction. Cervical, breast, and endometrial cancers were the most frequently associated with sexual dysfunction^22^. However, the anatomical location of the tumor is not the only cause of sexual dysfunction in women with cancer as it might also be induced by physiological changes from side effects of cancer treatments^22^. Reports of gynecological problems including decreased vaginal lubrication, sexual desire and arousal, and difficulty or inability to reach orgasm can result in sexual dysfunction^22,23^.

A meta-analysis of studies using the FSFI questionnaire conducted by Jing et al. (2019) also showed a higher incidence of sexual dysfunction in women with breast cancer (19.28; 95% CI 17.39–21.16) than in healthy women (FSFI > 26 normal sexual function)^25^. These results corroborate our findings, in which mean FSFI total scores in the exposed group were lower than the cutoff suggested by Wiegel et al. (2005), thus evidencing sexual dysfunction.

In this study, most women from the exposed group underwent conservative surgery, followed by chemotherapy and radiotherapy, and, less frequently, hormone therapy. Although treatment side effects might lead to sexual dysfunction, our study showed that exposure to cancer alone is sufficient to increase the risk for sexual dysfunction. This finding might be explained by the fact that psychological factors are involved in the etiology of sexual dysfunction^26^. Thus, cancer diagnosis and its related fears and apprehensions may affect sexual domains, including sexual desire, arousal, orgasm, and satisfaction.

Surgical cancer treatment may also change body image^22,24^ and prompt sexual dysfunction^23^. A prospective study conducted by Aerts et al. (2014) showed that women diagnosed with breast cancer had sexual dysfunction regardless of their surgical procedure (mastectomy or conservative). Moreover, symptoms in women undergoing mastectomy lasted longer (six months to one year after treatment completion) than in healthy women^24^. Mayer et al. (2018) also observed less sexual satisfaction and lower frequency of sexual activity, in addition to increased sexual discomfort, as reported by women with breast cancer^23^. These study results support our findings of increased chances of sexual dysfunction among the exposed group.

Sexual function is influenced by psychological and emotional factors, in addition to the endocrine (e.g., androgen, estrogen, progesterone, and prolactin), neuromusculoskeletal (e.g., pelvic floor muscles, fascias, and nerves), and vascular systems, each playing a part in different phases of sexual response^25,27^. The process of illness from cancer and its treatment can also lead to behavioral changes that influence sexual response, such as a reduction in the frequency of sexual activity^23^. Women in the exposed group had lower scores in sexual desire and lubrication and higher scores in sexual satisfaction and pain domains of the FSFI. Comparison between groups indicated better scores among the non-exposed group. Therefore, our findings support the hypothesis that breast cancer and its treatments may comprehensively interfere with the physiological function of body systems^24^.

The analysis of FSFI domains was also performed by Masjoudi et al. (2019)^28^ in a systematic review of studies including women with breast cancer. The authors showed that the lower scores referred to the sexual desire (2.8) and lubrication (2.8) domains, and the highest score referred to sexual satisfaction (5.09) domain, corroborating our findings. In a prospective longitudinal study, Jensen et al. (2003) observed reduced sexual desire, lubrication, altered vaginal morphology, and dyspareunia in women with cervical cancer compared with healthy women^29^. Women after mastectomy reported less sexual desire, arousal, and orgasm than before the surgery and showed more sexual dysfunction when compared with healthy women^24^.

The presence of comorbidities was analyzed in the final model of our study and appeared to increase the chances of sexual dysfunction. Almost half of our participants had chronic diseases, including hypertension, diabetes, arthritis, arthrosis, and dyslipidemia. Regardless of the cancer diagnosis, we observed that diabetes and hypertension were the main contributors to a higher risk of developing sexual dysfunction. Diabetes may lead to neurological impairments and vascular and psychological changes that may affect sexual desire, arousal, lubrication, orgasm and contribute to dyspareunia ^30–32^. Similarly, cardiovascular disease may favor atherosclerosis in the pelvic region arteries, reducing vaginal engorgement and contributing to clitoral erectile insufficiency syndrome, impairing lubrication, and diminishing sexual satisfaction^33^.

Menopause was not a significant variable in this study, but the literature cites as a relevant factor. Chemotherapy and hormone therapy, especially when administered in the pre-and perimenopause, contribute to hormonal changes that may trigger dryness and early vaginal atrophy and induce a sudden onset of menopause ^34,35^. More than half of the participants reported being in menopause. However, the stratified analysis indicated that most women in the exposed group were in menopause, which was not the case in the non-exposed group. This might explain the lack of significance of menopause in the final model.

In a prospective study, Abashe et al. (2009) observed that healthy women had better sexual function than women diagnosed with breast cancer who received hormone therapy. the latter group had better sexual function than women who received chemotherapy and radiotherapy^36^. Despite cancer and its treatment, the authors observed that menopause significantly reduced estrogen, negatively influencing mucosa vascularization, vaginal lubrication, and libido. This may trigger dyspareunia, lubrication dysfunction, and hypoactive desire throughout cancer treatment^37^.

We chose to add age in the adjusted model because of its reported importance on sexual dysfunction. Although our sample was between 45 and 50 years old, no interaction was observed between age and sexual dysfunction, which might be explained by the sample size. Among the general population, sexual dysfunction increases with age and is predominant in the reproductive phase^38^. Compared to healthy women, women exposed to cancer had lower sexual activity with increased age, suggesting a physiological effect of ageing^35^. Furthermore, young women with cancer showed a high incidence of sexual dysfunction related to body image and treatment type^39^.

One of the limitations of the study includes memory bias, which may have influenced the answers provided during the interviews. Also, we faced difficulties in accessing the participants by telephone due to the COVID-19 pandemic. Furthermore, patients were evaluated in different survival phases, both in the short survival phase (closer to the end of the oncological treatment) and in the long survival phase (5 years after diagnosis), which could modify the way the individual perceives the symptoms assessed. Therefore, these results should be interpreted with caution. Lastly, although we observed a considerable difference in the mean age between groups, this variable was added as an adjusted variable in the multivariate analysis and was insignificant in the final model.

This study has practical implications and highlights the importance of a multidisciplinary follow-up of women with sexual dysfunction. A clinical evaluation considering our findings may inform effective interventions. Moreover, early diagnosis of sexual dysfunction improves therapeutic outcomes. Our findings contribute to the body of evidence supporting the development of public policies for women with breast cancer in Brazil and countries with similar sociocultural and economic characteristics.

## Conclusion

This study showed that women diagnosed and treated for breast cancer had a higher incidence of sexual dysfunction than healthy women. Therefore, breast cancer was a risk factor for sexual dysfunction. Comorbidities also increased the chances of sexual dysfunction regardless of exposure to breast cancer.

## Data Availability

The datasets produced and examined in this study are not publicly accessible due to patient privacy concerns. However, interested parties can obtain access to these datasets by contacting the corresponding author through reasonable requests.

## Abbreviations

FSFI: Female sexual function index
AIC: Akaike information criterion
OR: Odds ratio
